# Rc3h1 negatively regulates osteoclastogenesis by limiting energy metabolism

**DOI:** 10.7150/thno.99565

**Published:** 2024-11-04

**Authors:** Liuyuan Chen, Yuangang Su, Chaofeng Wang, Qian Huang, Weiwei Chen, Na Hai, Jikang Wang, Haoyu Lian, Jinmin Zhao, Jiake Xu, Qian Liu

**Affiliations:** 1 Guangxi Key Laboratory of Regenerative Medicine, Orthopaedic Department, The First Affiliated Hospital of Guangxi Medical University, Nanning, Guangxi 530021, China.; 2 Collaborative Innovation Centre of Regenerative Medicine and Medical BioResource Development and Application Co-constructed by the Province and Ministry, Life Sciences Institute, Guangxi Medical University, Nanning, Guangxi 530021, China.; 3 Faculty of Pharmaceutical Sciences, Shenzhen University of Advanced Technology, and Chinese Academy of Sciences, Shenzhen, China.; 4 School of Biomedical Sciences, The University of Western Australia, Perth, Western Australia, Australia.

**Keywords:** Rc3h1, osteoclast, Tfr1, mitochondria, osteoporosis

## Abstract

**Rationale:** Osteoclasts are giant bone-resorbing cells that need vigorous mitochondrial respiration to support their activation. Rc3h1, an RNA-binding protein, precisely governs the homeostasis of mRNA. However, the precise role of Rc3h1 in regulating iron metabolism and mitochondrial respiration in osteoclasts is not yet understood.

**Methods:** We generated Rc3h1-deficient mice in osteoclast precursors and mature osteoclasts. The bone mass and osteoclast activity in bone tissues were evaluated. Moreover, we assessed the differentiation, bone resorption, iron content, and mitochondrial function of osteoclasts *in vitro*. In the end, the target gene of Rc3h1 and its role in mediating the effect of Rc3h1 on mitochondrial respiration in osteoclasts were further investigated.

**Results:** Mice lacking Rc3h1 exhibit low bone mass. In addition, Rc3h1 deletion in osteoclasts significantly promotes osteoclast activation. Mechanistically, Rc3h1 post-transcriptionally represses the expression of transferrin receptor 1 (Tfr1), restricting iron absorption and mitochondrial respiration in osteoclasts. Inhibition of Tfr1 in Rc3h1-deficient osteoclasts diminishes excessive osteoclast formation and mitochondrial respiration.

**Conclusion:** These findings suggest that Rc3h1 has a negative effect on osteoclast activation via limiting iron resorption and mitochondrial respiration. Finally, targeting the Rc3h1/Tfr1 axis might represent a potential therapeutic approach for bone-loss diseases.

## Introduction

Bone is a metabolically dynamic organ maintained by continuous bone renewal, completed by osteoclasts (OCs)-mediated old bone resorption and subsequent new bone formation by osteoblasts 1. Bone resorption exceeds bone formation in bone remodeling, caused by over-activated osteoclasts, resulting in bone mass loss. With the stimulation of two master regulators, macrophage colony-stimulating factor (M-CSF) and receptor activators of nuclear factor kappa-Β ligand (RANKL), bone marrow-derived macrophages (BMMs) differentiate into tartrate-resistant acid phosphatase (TRAP)-positive mononuclear cells and fuse to multinucleated osteoclasts, meanwhile migrating to the bone surface to resorb old bone 2. This process needs constant energy supply and metabolic reprogramming to meet osteoclast formation and bone resorption 3,4. Mitochondrial oxidative phosphorylation (OXPHOS) is the main source of energy supply in osteoclast differentiation 5. OXPHOS is positively associated with osteoclast differentiation, and targeting pathways modulating OXPHOPS might serve as an attractive target to preserve bone mass 6-8.

Rc3h1, also known as Roquin1, is an RNA-binding protein that works directly by binding with certain stem-loop structural elements in the 3' untranslated regions (3'-UTR) of mRNA 9,10. Rc3h1 is widely recognized as a regulator of T cell fates by suppressing the expression of key genes that play a critical role in Th1, Th17, and Tfh activation at the post-transcriptional level 11-13. Rc3h1 in macrophages specifically identifies the stem-loop motif in the 3'-UTR of TNF-α mRNA, inhibiting the synthesis of TNF-α 10. Rc3h1 could be seen as a 'gatekeeper' that limits immoderate immune responses and inflammatory processes. Also, Rc3h1 serves as a regulator for maintaining the homeostasis of micro-RNA levels 14. Additionally, Rc3h1 functions as an E3 ligase and involves the ubiquitinated degradation of the α1 subunit of adenosine monophosphate-activated protein kinase (AMPK) through its RING domain 15. Rc3h1 was reported to be a major regulator for cellular iron homeostasis by limiting the expression of transferrin receptor 1 (Tfr1), encoded by TFRC 16. The absence of Tfr1 in osteoclasts reduces mitochondrial biogenesis and OXPHOS by lowering Tfr1-mediated iron absorption 17. Furthermore, higher iron uptake promotes mitochondrial biogenesis and function in osteoclasts or adipocytes 18-20. Nevertheless, the role of Rc3h1 in regulating iron metabolism and mitochondrial respiration in osteoclasts remains unclear.

This work involved the deletion of Rc3h1 in both osteoclast precursors and mature osteoclasts to investigate the effect of Rc3h1 on bone mass and osteoclast formation. It was found that the absence of Rc3h1 led to significant bone loss, accompanied by an increased number of osteoclasts in bone tissues. Furthermore, Rc3h1 post-transcriptionally inhibits the expression of Tfr1, subsequently contributing to attenuated iron absorption and OXPHOS. Taken together, our findings reveal Rc3h1 as a novel regulator in controlling iron metabolism and mitochondrial respiration in osteoclasts.

## Results

### Deletion of Rc3h1 in osteoclast precursors leads to low bone mass

**Figure 1:**
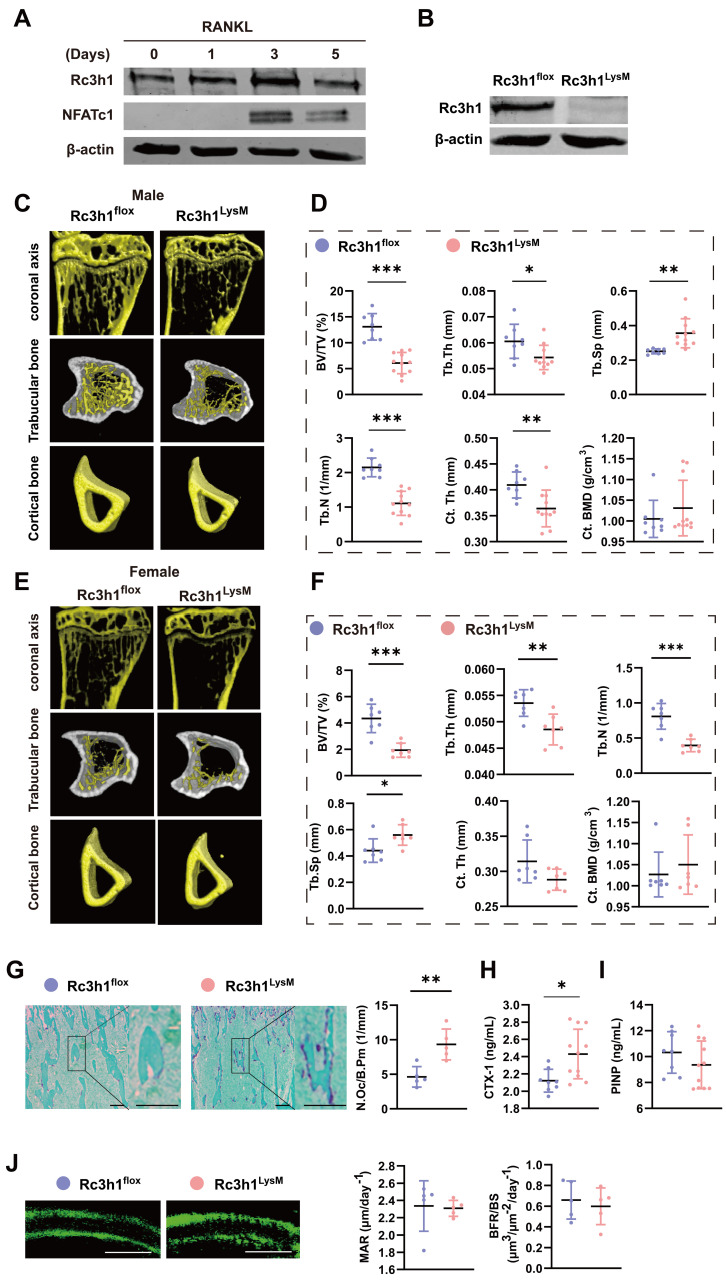
Low bone mass was exhibited in Rc3h1^LysM^ mice. (A) Protein level of Rc3h1 during osteoclast formation induced by M-CSF and RANKL from C57BL/6J mice. (B) The protein expression of Rc3h1 in Rc3h1^flox^ and Rc3h1^LysM^ osteoclasts. (C-D) Representative micro-CT images and quantitative analysis of proximal tibia trabecular and cortical bone in 12-week-old male Rc3h1^flox^ (n = 8) and Rc3h1^LysM^ mice (n = 11). (E-F) Representative micro-CT images and quantitative analysis of proximal tibia trabecular and cortical bone in 12-week-old female Rc3h1^flox^ (n = 7) and Rc3h1^LysM^ (n = 7) mice. (G) TRAP staining and analysis of proximal tibia from Rc3h1^flox^ (n = 5) and Rc3h1^LysM^ (n = 5) mice. Scale bar= 200 μm. (H-I) The ELISA of CTX-1 and PINP from Rc3h1^flox^ (n = 8) and Rc3h1^LysM^ (n = 11) serum. (J) Double calcein label of proximal tibia bone in Rc3h1^flox^ (n = 5) and Rc3h1^LysM^ (n = 5) mice. Scale bar= 50 μm. *P < 0.05, **P < 0.01, ***P < 0.001.

We evaluated the expression of Rc3h1 in the bone tissue of the femoral head of postmenopausal osteoporosis patients using the dataset GSE230665 21. The level of Rc3h1 expression was elevated in postmenopausal osteoporosis patients (PMOPs) compared to healthy postmenopausal women (Figure S1A-B). In addition, the western blot analysis revealed an upregulation of Rc3h1 during *in vitro* osteoclast formation induced by M-CSF and RANKL, as shown in Figure 1A. These results suggested that Rc3h1 potentially plays an important role in osteoporosis.

Our team has previously discovered that mice with a point mutation of Rc3h1 develop significant bone loss and increased osteoclasts in bone tissue 22. To elucidate the role of Rc3h1 in osteoclasts and bone diseases, we obtained Rc3h1 conditional knockout mice in osteoclast precursors by crossing Rc3h1-flox mice, where the exon 3 of the Rc3h1 gene was flanked with two loxP sites, with myeloid-specific LysM-Cre mice. Genotyping and WB analysis were used to identify wild-type (Rc3h1^flox/flox^; LysM-Cre^-^ referred to as Rc3h1^flox^) and knockout mice (Rc3h1^flox/flox^; LysM-Cre^+^ referred to as Rc3h1^LysM^), and to validate the knockout efficiency of Rc3h1 (Figure S1C and Figure 1B). Generally, Rc3h1^LysM^ mice did not show marked differences in body size and weight compared to WT littermates (data not shown). Micro-CT analysis of the proximal tibia of 12-week-old WT and Rc3h1^LysM^ mice revealed that deletion of Rc3h1 resulted in significant trabecular bone loss, reaching an approximately 2-fold reduction in BV/TV in both male and female mice compared to WT mice (Figure 1C-D and Figure 1E-F). In addition, the number of trabeculae (Tb.N) and the thickness of trabeculae (Tb. Th) were also significantly attenuated, while the trabecular separation (Tb. Sp) was enlarged in Rc3h1^LysM^ mice. Only male mice exhibited reduced cortical bone thickness (Ct. Th). The cortical bone mineral density (Ct. BMD) in Rc3h1^LysM^ mice was not noticeably altered in both genders.

Bone histological analysis revealed that the proximal tibial bone surface of Rc3h1^LysM^ was stained for more TRAP activity (Figure 1G), a marker of osteoclasts. Serum CTX-1 in Rc3h1^LysM^ mice was also increased (Figure 1H). The elevated osteoclast activity might affect the differentiation of osteoblasts 23, 24. However, the serum PINP levels, mineral deposition rate (MAR), and bone formation rate (BFR/BS) in Rc3h1^LysM^ mice were similar to those in the WT group (Figure 1I and 1J). These results showed that the loss of bone mass in Rc3h1-deficient mice may be attributable to enhanced osteoclast activity rather than osteoblast activity.

### Loss of Rc3h1 in differentiated osteoclasts results in decreased bone mass

To further explore the role of Rc3h1 specifically in differentiated osteoclasts, the Rc3h1^flox/flox^; Ctsk-Cre^-^ (Rc3h1^flox^) and Rc3h1^flox/flox^; Ctsk-Cre^+^ (Rc3h1^Ctsk^) mice were generated. The distal femurs were harvested from 12-week-old Rc3h1^flox^ and Rc3h1^Ctsk^ mice, and the bone tissues were subsequently scanned by Micro-CT.

**Figure 2:**
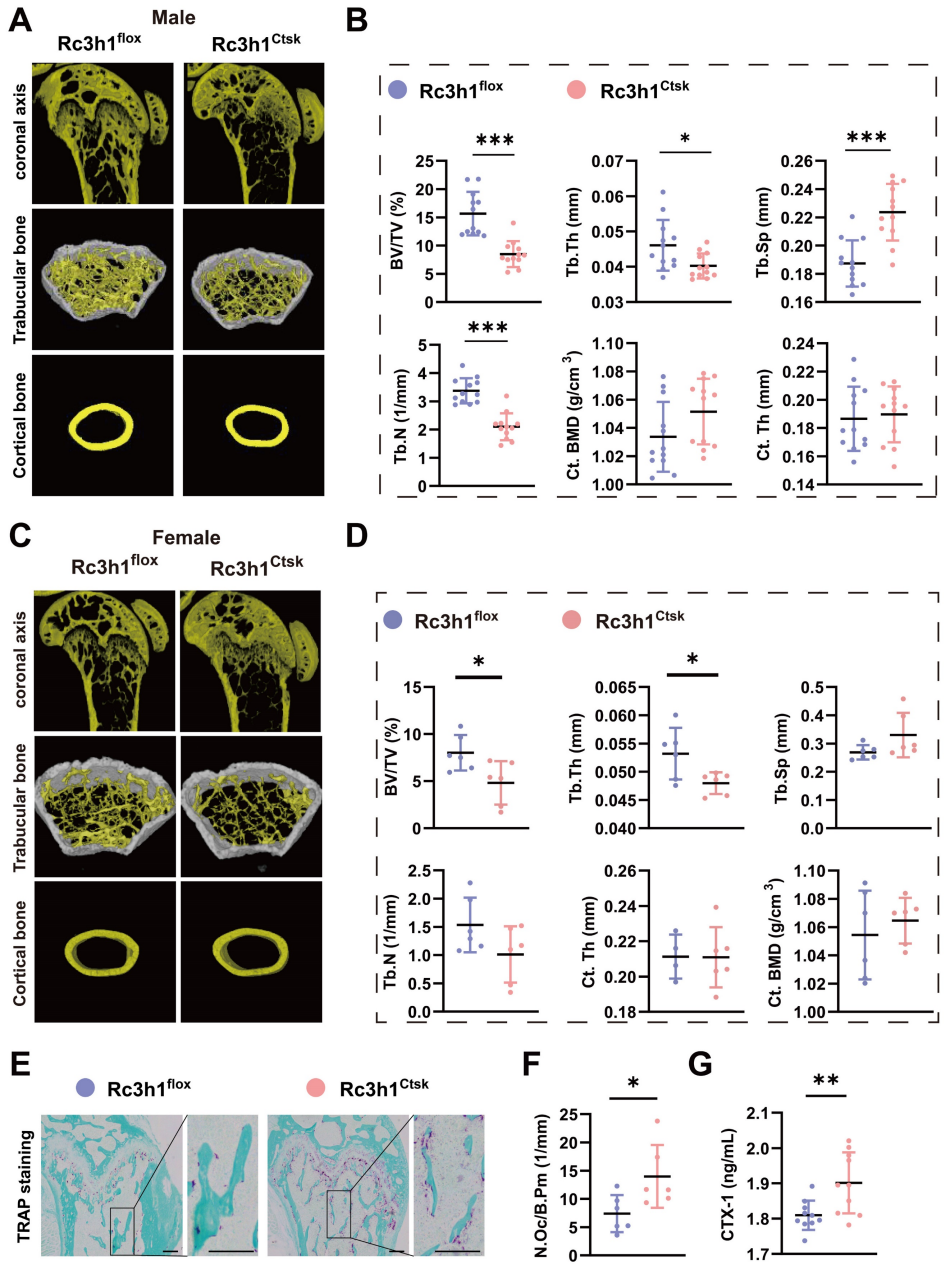
Deletion of Rc3h1 in differentiated osteoclasts leads to bone loss. (A-B) Representative 3D micro-CT reconstruction images and quantitative analysis of distal femur trabecular and cortical bone in 12-week-old male Rc3h1^flox^ (n = 12) and Rc3h1^Ctsk^ (n = 12) mice. (C-D) Micro-CT scanning and analysis of distal femur trabecular and cortical bone in 12-week-old female Rc3h1^flox^ (n = 6) and Rc3h1^Ctsk^ (n = 6) mice. (E-F) TRAP staining was used to quantitatively analyze the number of osteoclasts in distal femur bone tissue in 12-week-old male Rc3h1^flox^ (n = 6) and Rc3h1^Ctsk^ (n = 6) mice. (G) The serum CTX-1 was measured by ELISA. Scale bar= 200 μm (n = 10). *P < 0.05, **P < 0.01, ***P < 0.001.

As shown in Figure 2A-D, the BV/TV and Tb. Th were decreased in male and female Rc3h1^Ctsk^ mice. The Tb. N was declined, and the Tb. Sp was increased only in male Rc3h1^Ctsk^ mice. The Ct. Th and Ct.BMD were not altered in both genders of Rc3h1^Ctsk^ mice compared to their WT littermates. Consistent with the Micro-CT findings, the TRAP staining of the distal femur from Rc3h1^Ctsk^ demonstrated more osteoclast activity (Figure 2E-F). The serum CTX-1 level in Rc3h1^LysM^ mice also was increased (Figure 2G).

### Rc3h1 deficiency facilitates the formation of osteoclasts

**Figure 3:**
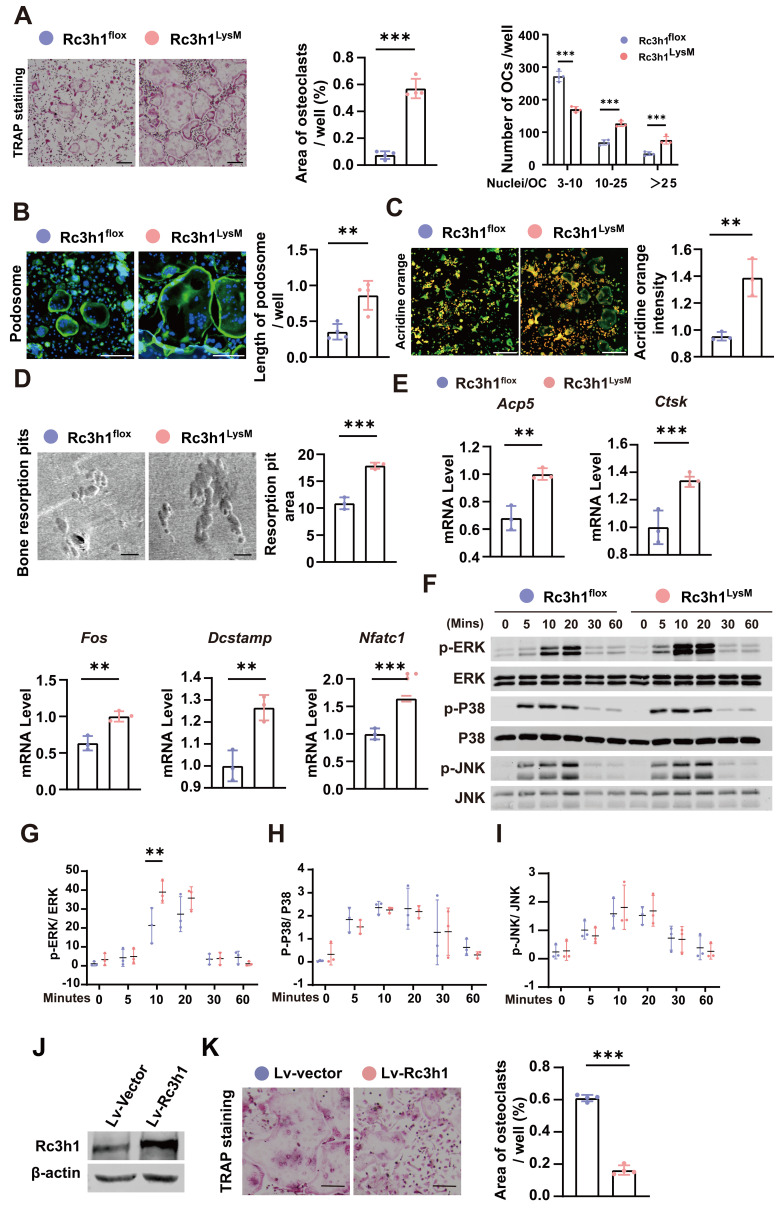
Loss of Rc3h1 accelerates osteoclast formation. (A) *In vitro* TRAP staining was performed on osteoclasts from Rc3h1^flox^ and Rc3h1^LysM^ mice. Scale bar= 200 μm. (B) Podosome staining on Rc3h1^flox^ and Rc3h1^LysM^ osteoclasts. Scale bar= 200 μm. (C) Representative images of acidification in osteoclasts from Rc3h1^flox^ and Rc3h1^LysM^ osteoclasts. Scale bar= 300 μm. (D) Representative images of scanning electron microscopy on bone resorption pits from Rc3h1^flox^ and Rc3h1^LysM^ osteoclasts. Scale bar= 100 μm. (E) Expression levels of osteoclast-related genes on BMMs with the induction of M-CSF and RANKL for 3 days assessed using qPCR. (F-I) Proteins and phosphorylation states of ERK, P38, and JNK upon the stimulation of RANKL. (J-K) The WB of Rc3h1 and TRAP staining of osteoclasts stimulated with RANKL and M-CSF for 6 days from BMMs transfected with Lv-vector or Lv-Rc3h1. *P < 0.05, **P < 0.01, ***P < 0.001.

BMMs were isolated from the tibias and femurs of Rc3h1^flox^ and Rc3h1^LysM^ mice and cultured with M-CSF and RANKL to generate mature osteoclasts. As shown in Figure 3A-B, Rc3h1^LysM^ osteoclasts demonstrated a more robust capability to spread and form larger morphologies of mature osteoclasts and F-actin rich podosome-belts. There was an increase in the activation of NFATc1 and its translocation from the cytoplasm to the nucleus (Figure S2A-B). Rc3h1 deletion enhanced the capacity of osteoclasts to acidify the extracellular circumstances and degrade the bone matrix, as shown by higher acid content and more bone resorptive pits (Figure 3C and D). The expression of genes, including *Acp5, Ctsk, Fos, Dc-stamp,* and* Nfatc1*, required for osteoclast differentiation, was also increased in Rc3h1^LysM^ osteoclasts compared to controls (Figure 3E). The protein levels of CTSK, NFATc1, ACP5 and c-FOS were also raised in Rc3h1-deficient osteoclasts (Figure S2C-D). Furthermore, deficiency of Rc3h1 overly activated the RANKL-induced ERK signaling pathway (Figure 3F-I). We simultaneously overexpressed Rc3h1 in BMMs and induced osteoclasts in M-CSF and RANKL until mature osteoclasts formed in controls. The expression of Rc3h1 was evaluated in control and Rc3h1-overexpressed osteoclasts (Figure 3J). We observed a decrease in osteoclastogenesis in Rc3h1-overexpressed osteoclasts compared to the control group, which had already formed mature and large osteoclasts (Figure 3K). These results indicate that loss of Rc3h1 fuels osteoclast formation and function in an intrinsic manner.

### Rc3h1 targets the mRNA of Tfr1 and restrains Tfr1-mediated iron resorption in osteoclasts

**Figure 4:**
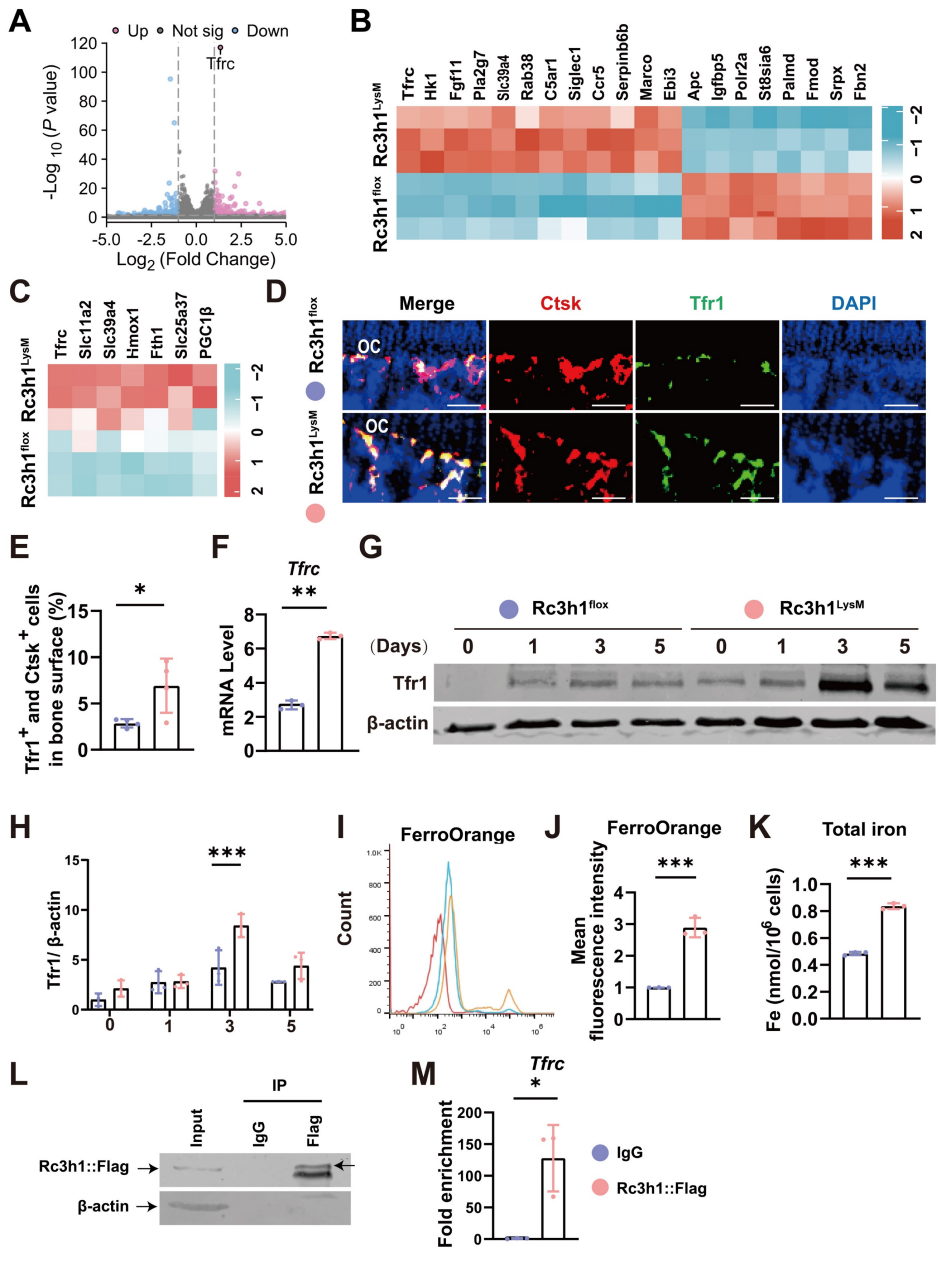
Tfr1 is a candidate target of Rc3h1 in osteoclasts. (A-B) Volcano plot and heatmap of differentially expressed genes in Rc3h1^flox^ and Rc3h1^LysM^ osteoclasts induced by M-CSF and RANKL for 3 days (n = 3). (C) Heatmap of iron metabolism-related genes in Rc3h1^flox^ and Rc3h1^LysM^ osteoclasts. (D-E) Immunofluorescent staining and quantitative analysis of Tfr1 on proximal tibia bone slides from Rc3h1^flox^ and Rc3h1^LysM^ mice (n = 4). Scale bar= 250 μm. (F) mRNA level of Tfr1 using qPCR in WT and Rc3h1-deficient osteoclasts. (G-H) Protein level of Tfr1 during the formation of osteoclasts in WT and Rc3h1-deficient osteoclasts. (I-J) Results of mean fluorescent intensity of FerroOrange from WT and Rc3h1-deficient osteoclasts using flow cytometry to detect cellular Fe^2+^content. (K) Total iron from lysed otseoclasts measured by absorbance at 593 nm. (L-M) Western bolts were presented to confirm the Rc3h1-IP efficiency, and qPCR was used to quantify the indicated mRNA in the IP and IgG groups. RIP was performed on RAW264.7 cells transfected with the Rc3h1-Puro-Flag vector after RANKL stimulation for 3 days (n= 3). *P < 0.05, **P < 0.01, ***P < 0.001.

As an RNA-binding protein, Rc3h1 promotes the mRNA of target genes decay by directly binding the 3′-untranslated region of mRNA 10,25. Rc3h1 targets the mRNA of Icos and Ox40 in follicular helper T cells, cell cycle-promoting genes in breast cancer cell lines, and Tfr1 in HAP1, HUVEC, L-M, and MEF cell lines 16,26,27, suggesting Rc3h1 controls the abundance of mRNA in a cell-specific manner. Subsequently, we utilized RNA-seq to identify possible targets of Rc3h1 in osteoclasts from Rc3h1^LysM^ and WT mice. As shown in Figure 4A-B, *Tfrc*, which encodes the Tfr1 protein, is the most significantly upregulated gene. A previous study has found that Rc3h1 is a major mediator of Tfr1-mediated iron metabolism, and the mRNA is one of the targets of Rc3h1 in HAP1, HUVEC, L-M, and MEF cell lines 16. Tfr1 is widely recognized as the primary carrier of iron and plays an essential role in regulating cellular iron homeostasis 28,29. Thus, we focused on the iron metabolism-related genes and found these genes were upregulated (Figure 4C). Next, the expression of Tfr1 on osteoclasts from bone tissues was higher in Rc3h1^LysM^ than in WT littermates (Figure 4D-E). Consistently, we verified the increased mRNA level of Tfr1 (Figure 4F) and the protein level of Tfr1 (Figure 4G-H) in Rc3h1^LysM^ osteoclasts. Meanwhile, the Fe^2+^ and total iron content in Rc3h1^LysM^ osteoclasts were significantly elevated (Figure 4I-K). Eventually, we confirmed the interaction of Rc3h1 with the mRNA of Tfr1 using an RNA immunoprecipitation (RIP) assay (Figure 4L and 4M). In conclusion, Rc3h1 negatively controls cellular iron content through limiting Tfr1-mediated iron resorption in osteoclasts.

### Rc3h1 negatively modulates mitochondrial respiration

**Figure 5:**
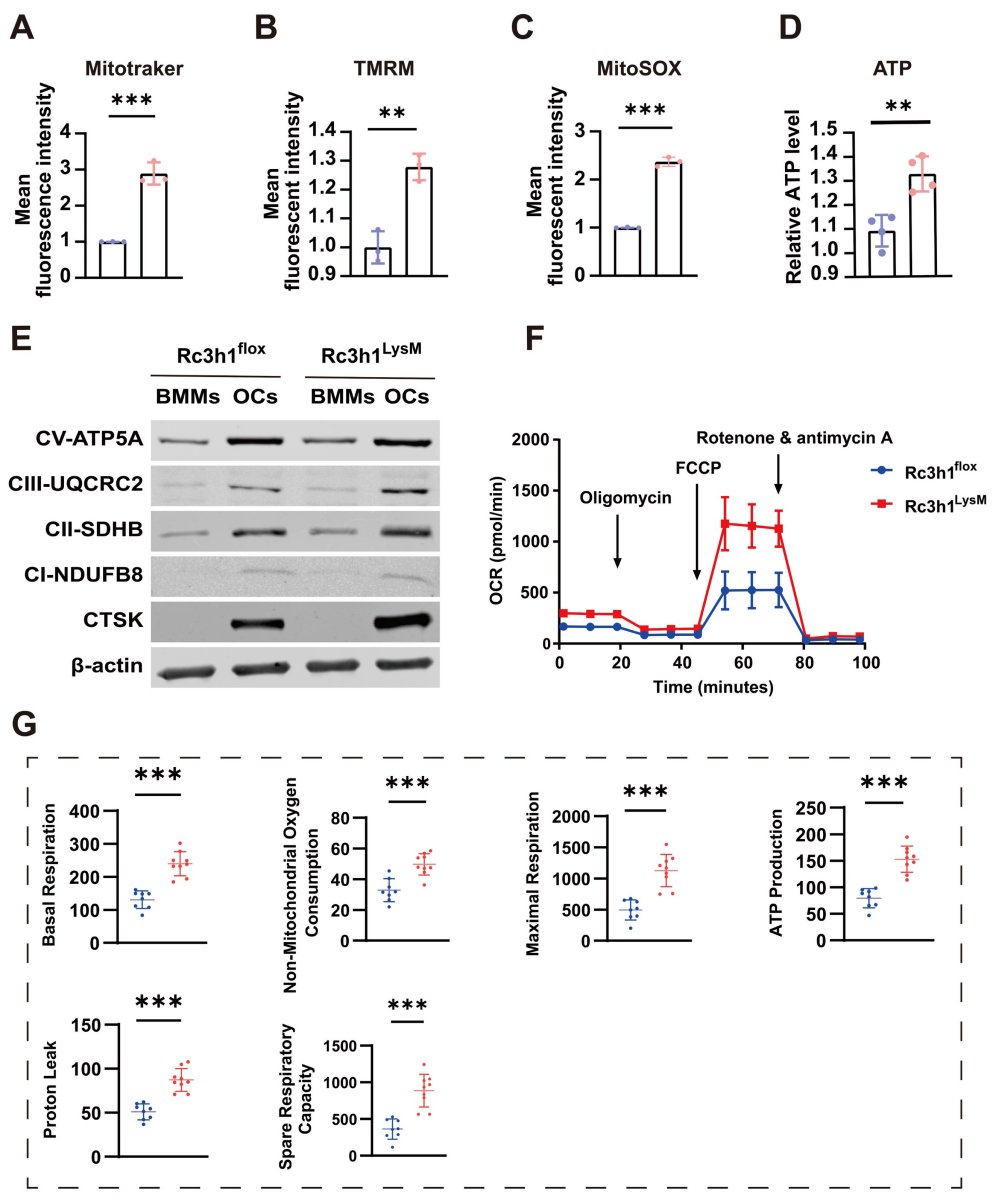
Loss of Rc3h1 in osteoclasts results in enhanced mitochondrial respiration. (A-C) Flow cytometry analysis of osteoclasts' mitochondrial mass, ROS, and membrane potential stained with mitogreen, mitoSOX, and TMRM, respectively. (D) Relative cellular ATP content in WT and Rc3h1-deficient osteoclasts. (E) Western blot evaluation of mitochondrial respiration complexes C-I to C-III, C-V and CTSK in Rc3h1^flox^ and Rc3h1^LysM^ osteoclasts. (F-G) Extracellular oxygen consumption rate (OCR) analysis and statistical analysis of Rc3h1^flox^ and Rc3h1^LysM^ osteoclasts using seahorse assay. All the osteoclasts were derived from Rc3h1^flox^ and Rc3h1^LysM^ BMMs after M-CSF and RANKL induction for 3 days. *P < 0.05, **P < 0.01, ***P < 0.001.

Tfr1-mediated iron absorption is crucial for mitochondrial OXPHOS 17, 19. The differentiation of osteoclast relies heavily on the energy obtained from OXPHOS 30, and modulation of mitochondrial OXPHOS also determines the fate of osteoclast 7,18. In Rc3h1-deficient osteoclasts, we observed a significant enhancement in Tfr1-mediated iron absorption. Firstly, we measured mitochondrial mass, membrane potential, and ROS levels in Rc3h1^LysM^ and Rc3h1^flox^ osteoclasts using flow cytometry. In Rc3h1^LysM^ osteoclasts, the mean fluorescent intensity of mitotraker, tetramethylrhodamine (TMRM), and mitoSOX was significantly increased (Figure 5A-C). The absence of Rc3h1 resulted in a higher production of ATP (Figure 5D). We then performed WB analyses to examine the expression of the OXPHOS complexes. A modest increase in Rc3h1^LysM^ osteoclasts was observed for complexes II and III (Figure 5E). Finally, the extracellular oxygen consumption rate (OCR) of Rc3h1-deficient osteoclasts and controls was assessed to measure mitochondrial respiration. Similarly, the Rc3h1^LysM^ osteoclasts displayed a higher level of extracellular oxygen consumption rate compared to Rc3h1^flox^ (Figure 5F-G). Our results indicated that Rc3h1 constraints mitochondrial respiration and ATP production in osteoclasts.

### Tfr1 mediates the effects of Rc3h1 on mitochondrial respiration in osteoclasts

**Figure 6:**
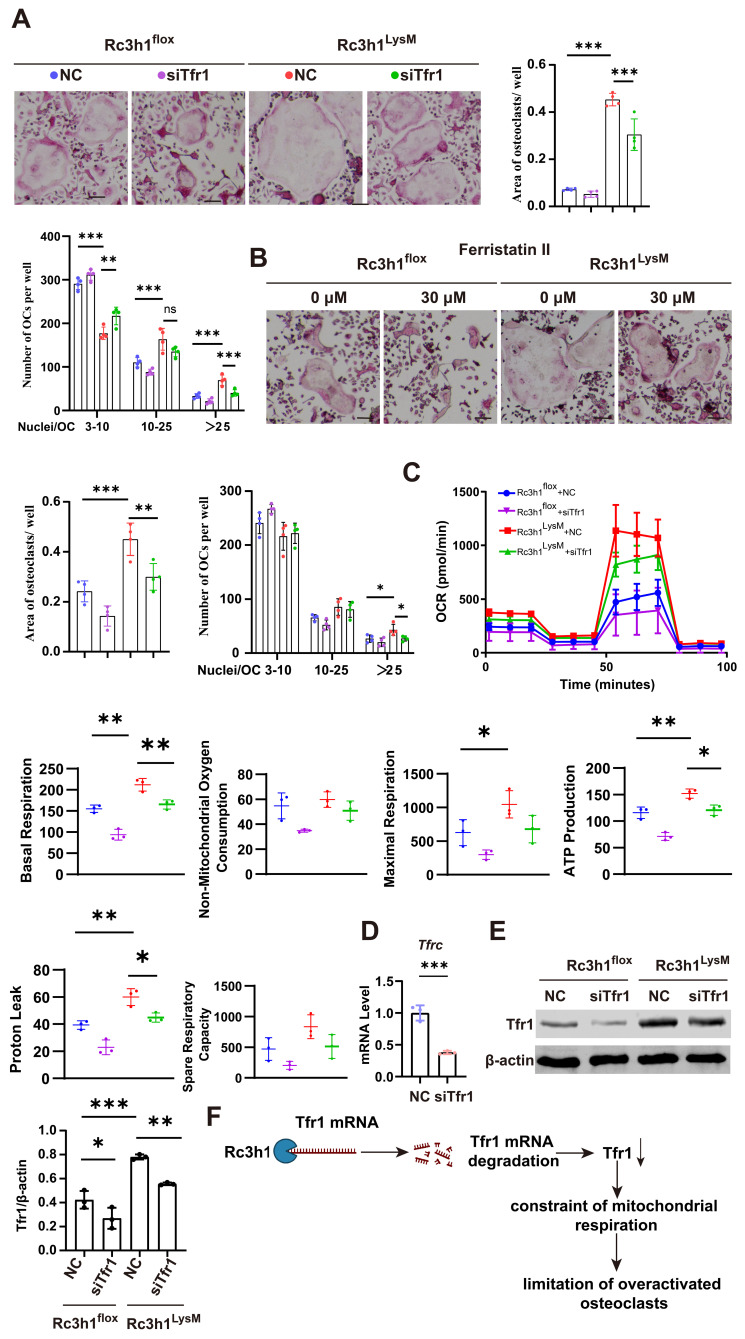
Tfr1 mediates the effects of Rc3h1 on osteoclastogenesis and mitochondrial respiration. (A-B) Representative TRAP staining images and quantitative analysis of osteoclasts induced by M-CSF and RANKL 5 days with the presence of siTfr1 or Ferristatin II. Scale bar= 200 μm. (C) OCR analysis of Rc3h1^flox^ and Rc3h1^LysM^ osteoclasts using seahorse assay with or without the presence of siTfr1. (D) The mRNA expression of Tfr1 in osteoclasts treated with negative control or siTfr1 using qPCR. (E) The protein expression of Tfr1 in osteoclasts treated with negative control or siTfr1. (F) The diagram of the mechanism of Rc3h1 regulating mitochondrial respiration in osteoclasts (created in BioRender.com). *P < 0.05, **P < 0.01, ***P < 0.001.

We investigated whether the elevated Tfr1 in Rc3h1-deficient osteoclasts mediates the enhanced mitochondrial respiration. To this end, the expression of Tfr1 was inhibited using either ferristatin II, a small-molecule inhibitor of Tfr1, or siRNA targeting Tfr1. Ferristatin II functions via facilitating the degradation of Tfr1 31. We observed that inhibition of Tfr1 by siRNA effectively mitigated the over-activated osteoclastogenesis induced by RANKL and M-CSF in Rc3h1^LysM^ BMMs, as depicted in Figure 6A. Similarly, the osteoclastogenesis of Rc3h1^LysM^ BMMs was reduced when treated with ferristatin II (Figure 6B). Simultaneously, the OCR in Rc3h1^LysM^ osteoclasts was decreased when Tfr1 was inhibited by siRNA (Figure 6C). Additionally, the mRNA level of Tfr1 after knockdown by siTfr1 was evaluated through qPCR (Figure 6D). The protein level of Tfr1 in Rc3h1^flox^ and Rc3h1^LysM^ osteoclasts treated with siTfr1 also was measured by WB (Figure 6E).

## Discussion

Over-activated osteoclasts lead to excessive bone resorption, resulting in a disruption of the bone remodeling balance, which consequently causes osteoporosis 32. Limiting the hyper-activation of osteoclasts has been one of the mainstream strategies for developing therapeutic agents for osteoporosis 33. Rc3h1 has been widely reported to limit the overwhelming activation of T cells 34, proliferation of breast cancer cells 27, and excessive production of inflammatory factors secreted by macrophages 10. It is reminiscent that Rc3h1 is likely capable of inhibiting the overt formation of osteoclasts. Indeed, a point mutation (M119R) in the Rc3h1 gene is associated with loss of bone mass in Sanroque mice 22. Initially, we found upregulated Rc3h1 expression in osteoclasts under the pathologic condition of osteoporosis. Osteoclast-specific deletion of Rc3h1 causes bone loss. *In vitro* osteoclast differentiation showed that deletion of Rc3h1 in osteoclasts led to hyperactivation of osteoclasts. Mechanistically, Rc3h1 could target Tfr1 mRNA and promote its degradation, reducing cellular iron uptake and preventing excessive amplification of mitochondrial respiration, consequently repressing osteoclast hyperactivation.

The most exciting finding in our study might be the low bone mass phenotype in Rc3h1^LysM^ mice. This phenotype is mainly caused by excessive bone resorption by osteoclasts, leading to imbalanced bone remodeling. Likewise, deletion of Rc3h1 accelerates osteoclast formation during RANKL induction *in vitro*, explaining the low bone mass phenotype in mice. Several critical coupling factors secreted by osteoclasts promote new bone formation by osteoblasts 35. This coupling mechanism appears to not work in Rc3h1-deficient mice, where excessive bone resorption does not significantly affect new bone deposition. Another interesting phenomenon is that after the deletion of Rc3h1, major bone loss occurs in the trabecular bone region, with no significant change in cortical bone density. Trabecular and cortical bone exhibit distinct structural properties, metabolic activity, and rates of bone turnover 36,37. Given the larger surface area available for bone remodeling in trabecular bone, the trabecular bone is the leading site where bone loss occurs during the initial phases of osteoporosis 38. This may account for the notable reduction of bone mass in trabecular bone rather than cortical bone in Rc3h1-deficient mice.

Trabecular bone mass was reduced in both male and female Rc3h1^LysM^ and Rc3h1^Ctsk^ mice. Nevertheless, this phenotype appears to be more prominent in male mice in comparison to female mice, particularly when Rc3h1 is deleted in mature osteoclasts. It is wildly accepted that the drop in the estrogen level upon menopause could result in bone loss and overactivated osetoclastogenesis. In osteoclasts, estrogen suppresses the expression of Tfr1, as well as mitochondrial function and energy metabolism 17, 39.

Rc3h1 has been characterized as an RNA-binding protein that functions as a transcriptional repressor by facilitating the degradation of the mRNA of target genes 10,40. As previously mentioned, Rc3h1 harbors a broad pool of target genes and selects its targets in a context- and cell-dependent manner 25,26,27. Recently, Corral, VM, *et al.* reported that Rc3h1 regulates cellular iron metabolism by targeting and destabilizing the mRNA of Tfr1 in four different cell lines (HAP1, HUVEC, L-M, and MEF) 16. Using RNA-seq, Tfr1 was the most significantly differential gene, and genes related to iron metabolism were also significantly altered, enlightening us to select Tfr1 as a candidate target of Rc3h1 in osteoclasts. Subsequent experiments revealed a markedly higher expression of Tfr1 and an elevated iron content in Rc3h1-deficient osteoclasts. Meanwhile, the RIP assay verified the capacity of Rc3h1 binding with the mRNA of Tfr1, substantiating that Tfr1 is one of the targets of Rc3h1 in osteoclasts.

Tfr1-mediated iron uptake is the predominant approach for the cellular acquisition of iron 41. Tfr1 and iron are strongly associated with mitochondrial function in osteoclasts and other cells, mainly contributing to the facilitation of mitochondrial iron-sulfur cluster synthesis, heme synthesis, and the efficiency of electron transport 18,20,42. Given the significantly increased Tfr1 and iron in Rc3h1-deleted osteoclasts, it is plausible to infer that mitochondrial metabolism might be strengthened. We found that mitochondrial mass, membrane potential, ROS, and extracellular oxygen consumption are elevated in Rc3h1-deficient osteoclasts. There are discrepancies between the results of the protein levels of mitochondrial complexes and other indicators of mitochondrial metabolism revealed by flow cytometry. Bhaba K. Das1 reported that the knockout of Tfr1 in osteoclasts decreased mitochondrial complexes II and III without affecting I, IV, and V 17. One possible explanation is that the differential changes of subunits of ETC might be attributed to the varying distribution of iron-sulfur clusters or heme in the subunits of the electron transport chain 43. An additional critical phenotype is that Rc3h1 deficiency leads to hyperactivation of osteoclasts. NFATc1, a major transcription factor that determines osteoclast differentiation, is phosphorylated in the cytoplasm, and then translocates to the nucleus to initiate transcriptional expression of osteoclast-related genes 44. Increased NFATc1 translocation into the nucleus in Rc3h1-deleted osteoclasts indicates enhanced osteoclast formation. Whether the alteration of mitochondrial function affects the number of osteoclasts is controversial. PGC1β is a major regulator of mitochondrial biogenesis and function in osteoclast 7. Zhang reported that loss of PGC1β in osteoclast precursor cells results in reduced mitochondrial biogenesis and respiration, leading to reduced osteoclast bone resorption without affecting osteoclast differentiation 45. Another piece of evidence shows that deletion of PGC1β in osteoclasts inhibits osteoclast differentiation and mitochondrial function 18. We also found that loss of Rc3h1 exhibited a larger cytoskeleton. It should be noted that counting the number of osteoclasts (TRAP-positive and nuclei ≥3) might underestimate the actual capacity of Rc3h1^LysM^ osteoclast differentiation, especially when compared to control osteoclasts. Thus, vigorous mitochondrial respiration is most likely responsible for osteoclast hyperactivation in Rc3h1^LysM^ osteoclasts. We found that the ERK signaling pathway was significantly enhanced in Rc3h1-deficient osteoclasts. Intracellular ROS originates mainly from the electron transport chain of mitochondria 46. Our findings suggest that loss of Rc3h1 promotes Tfr1-mediated iron uptake in osteoclasts and the subsequent mitochondrial respiration. The enhanced mitochondrial OXPHOS contributes more intracellular ROS generation. The activation of the ERK signaling pathway mediated by ROS has been well studied 47. A plausible explanation is that the increased activity of the ERK signaling pathway might be attributed to the augmented production of mitochondrial ROS.

In this study, we evaluate the essential role of Rc3h1 in osteoclast differentiation and function. The present study elucidates a post-transcriptional mechanism by which Rc3h1 regulates iron metabolism and mitochondrial respiration in osteoclasts. Our research suggests that Rc3h1 may be a therapeutic target for bone loss diseases.

## Materials and Methods

### Mice

The Rc3h1-flox (Rc3h1^flox^); LysM-Cre and Rc3h1-flox (Rc3h1^flox^); Ctsk-Cre mice were purchased from GemPharmatech Co., Ltd. (Nanjing, China). Briefly, we crossed Rc3h1^flox^ mice with LysM-Cre or Ctsk-Cre mice to obtain the myeloid-specific and differentiated-osteoclast Rc3h1 knockout mice. C57BL/6J mice were obtained from the animal center of Guangxi Medical University. All the mice were maintained at the animal center of Guangxi Medical University with a 12-hour light cycle, free access to water, and a standard laboratory diet. All the animal studies were approved and supervised by the committee's Animal Care and Welfare Committee of Guangxi Medical University (Experimental Ethics Approval Number: 202005177).

### Micro-computed tomography (Micro-CT) analysis

After removing the soft tissue of the tibia and femur from 12-week-old mice, the proximal tibia and distal femur were preserved overnight in 4% paraformaldehyde and washed three times with PBS before a micro-CT scan (SkyScan 1176, Bruker). The bone volume per tissue volume (BV/TV), trabecular number (Tb. N), trabecular space (Tb. Sp), trabecular thickness (Tb. Th), cortical bone mineral density (Ct. BMD), and cortical thickness (Ct. Th) were analyzed using the CTAn software (SkyScan). The 3D reconstruction of the tibia and femur was performed using CTvox software.

### Bone histomorphometry

The tibias were decalcified for 14 days and embedded in paraffin for sectioning with a 5 μm thickness, and TRAP staining was performed. Images were acquired by a KFPRO scanner and visualized by K-Viewer software (KONFOONG BIOTECH INTERNATIONAL CO., LTD., Ningbo, China). For Tfr1 detection, double fluorescent staining for Ctsk and Tfr1 was performed, and the preparation for cryosection of the tibia bone was completed with reference to previous literature 48. The fresh bone tissues were dissected, and the bone tissue was fixed in 4% paraformaldehyde for 24 hours immediately. Next, the bone tissues were decalcified in EDTA for 48 hours, and the EDTA was replaced with a cryoprotective solution (20% sucrose and 2% polyvinylpyrrolidone) 24 hours before embedding in the embedded solution (8% gelatin, 20% sucrose, and 2% PVP). After embedding, the bone tissues were preserved at -80 °C before section. The 50 μm thickness for the bone section was obtained. Following the permeabilization, the primary antibodies for Ctsk (sc-48353, Santa Cruz) and Tfr1 (sc-65882, Santa Cruz), followed by second antibodies, were used for the staining. The fluorescent images were acquired by the confocal microscope (Leica STELLARIS, Heidelberg, Germany). ImageJ software was used to analyze the bone histomorphometry.

### BMMs isolation and osteoclast culture

BMMs isolation was carried out with reference to the other research 7. Briefly, bone marrow cells were extracted from the tibia and femur, then lysed by the Red Blood Cell Lysis Buffer (C3702, Beyotime, China), and subsequently cultured in α-minimum essential medium plus 10% fetal bovine serum, 1% penicillin-streptomycin, and macrophage colony-stimulating factor. After 48 hours of culture, nonadherent cells were removed, and the complete media was refreshed. For osteoclast differentiation, BMMs were seeded into 96-well plates and induced by M-CSF and RANKL, and the medium containing M-CSF and RANKL was changed every other day to generate mature osteoclasts for 4-6 days. TRAP staining was conducted with a TRAP kit (Sigma-Aldrich). Cells positive for TRAP staining and ≥3 nuclei were counted as osteoclasts.

### Extracellular acidification assay and bone resorption assay

For detecting acid production, BMMs stimulated with RANKL for 3 days were stained with acridine orange. For bone resorption, BMMs were seeded on the bovine cortical bone and cultured with RNAKL for 9 days. The bovine cortical bones were harvested and scanned with a scanning electron microscope as reported 49.

### Lentiviral transfection

Rc3h1-overexpressing lentiviruses were purchased from Sangon Biotech (Shanghai) Co., Ltd. BMMs or RAW264.7 cells were infected with lentiviruses at a multiplicity of infection (MOI) of 50 or 150, respectively, with the help of 4 μg/mL polybrene. 48 hours later, the BMMs or RAW264.7 cells were screened with puromycin (4 or 10 μg/mL for BMMs and RAW264.7 cells respectively).

### Total RNA isolation and qPCR

BMMs were seeded in 6-well plates and induced with RANKL for 3-5 days. According to the manufacturer's instructions, TRIzol reagent was used to extract the total RNA, and the total RNA concentration was measured with NanoDrop One/One^C^ (ThermoFisher). Reverse RNA transcription to cDNA was done with a Reverse Transcription Kit (k16225, Thermo Fisher) using a 20 μL reaction system. A quantitative polymerase chain reaction was performed on a PCR instrument (Roche, Basel, Switzerland). Gene expression was calculated using the 2^-ΔΔCt^ method. The expression of the gene was normalized to the average expression level of *Actb*.

### Western blots

BMMs stimulated with RANKL for 3-5 days were lysed using RIPA buffer, which included a protease inhibitor, PMSF, and a phosphatase inhibitor. The lysates were then heated at 100 °C for 10 minutes and mixed with loading buffer. Proteins were loaded to 8%-15% SDS-polyacrylamide gel electrophoresis and transferred to polyvinylidene difluoride membranes. Then, the membranes containing proteins were blocked with 5% fat-free milk for 1 hour, followed by incubation with the primary antibody overnight at 4^ ◦^C. After incubation, the membranes were washed with 1× tris-buffered saline plus 0.05% Tween for 5 minutes three times and then incubated with the second antibody for 1 hour. Images were obtained using the Image Quant LAS-4000 system (GE Healthcare, Chicago, Illinois, USA). The Image J software was used to quantify the protein level. The primary antibodies are as follows: anti-Rc3h1 (#70195, Abcam), anti-CTSK (#sc-48353, Santa Cruz, USA), anti-NFATc1 (#sc-7294, Santa Cruz, USA), anti-Tfr1 (#sc-65882, Santa Cruz, USA), anti-ERK1/2 (#4695, cell signaling, CHINA), anti-p-ERK1/2 (#4370, cell signaling, CHINA), anti-p38 (#8690, cell signaling, CHINA), anti-p-p38 (#4511, cell signaling, CHINA), anti-JNK (#9252, cell signaling, CHINA), anti-p-JNK (#4668, cell signaling, CHINA), anti-CI-NDUFB8 (#ab110242, Abcam, UK), anti-CII-SDHB (#ab14714, Abcam, UK), anti-CIII-UQCRC2 (#ab14745, Abcam, UK), and anti-V-ATP5A (#ab14748, Abcam, UK).

### RNA-seq

BMMs were seeded into 6-well plates and stimulated with RANKL for 3 days. Total RNA was harvested using the TRIzol reagent. The RNA sequencing was completed using the Illumina HiseqTM platform by Sangon Biotech Co., Ltd. (Shanghai, China). The raw reads in FSTAQ format were passed through FastQC and subsequently mapped to the *Mus musculus* genome using HISAT2 software 50. The gene expression level was estimated based on TPM (transcript per million) with StringTie software, and differential genes were defined as Q values (Benjamini-Hochberg-adjusted P values) ≤ 0.05.

### RNA-immunoprecipitation (RIP)

Approximate 1×10^7^ RAW264.7 cells transfected with an Rc3h1-Flag-puromycin-expressing plasmid were stimulated with RANKL for 3 days. The RIP was performed according to the EZ-Magna RIP kit protocol (17-701, Merck, Sigma-Aldrich). Briefly, the cells were lysed by RIP lysis buffer and stored at -80 °C for less than 3 months for further use. Firstly, the magnetic beads were incubated with anti-Flag (390002, Zen-Bioscience, China) or IgG (17-701, Merck, Sigma-Aldrich) antibodies at room temperature for 30 minutes. Then, the lysates were incubated with magnetic beads at 4 °C overnight, and the magnetic beads were washed six times. Rc3h1-bound RNA purification was accomplished by the proteinase K buffer, followed by RNA extraction. Finally, the fold enrichment of RNA was analyzed by qPCR using the manufacturer's reference instructions.

### Cellular iron detection

The Fe^2+^ detection was performed in flow cytometry as described below. To measure the total iron content, BMMs were stimulated with RANKL for 3 days. The cells were lysed, and total iron was calculated using the Cell Total Iron Colorimetric Assay Kit (E-BC-K880-M, Elabscience, China) according to the manufacturer's instructions.

### Flow cytometry

For mitochondrial content, membrane potential, mitochondrial ROS detection, and Fe^2+^ detection, BMMs were seeded into 6-well plates and stimulated with RANKL for 3 days, then stained with the MitoTraker probes (M7514, ThermoFisher), TMRM (I34361, ThermoFisher), MitoSOX (M36008, ThermoFisher), or FerroOrange (F374, Dojindo, Japan) for 30 minutes at 37 °C according to the manufacturer's instructions. The cells were rinsed with PBS and lifted to be analyzed using Flow 6 (USA).

### Extracellular Oxygen Consumption Assay

Dynamic analysis of mitochondrial function was based on measurements of extracellular oxygen consumption rate in osteoclasts. BMMs were seeded into XFe24 plates and stimulated with RANKL for 3 days. 1.5 μM oligomycin, 2 μM FCCP, 0.5 μM rotenone/ antimycin A were sequentially injected into wells followed by detection of OCR using the Seahorse XFe24 Extracellular Flux Analyzer.

### ATP measurement

1×10^6^ BMMs were seeded in 6-well plates and cultured with RANKL and M-CSF for 3 days. The relative ATP content was measured with an ATP detection kit (G7571, Promega) according to the manufacturer's instructions.

### Inhibitor and siRNA of Tfr1

The inhibitor of Tfr1, Ferristatin II, was purchased from Sigma (C1144-5G). The siRNA for Tfr1 was designed and synthesized by Sangon Biotech Co., Ltd. (Shanghai, China). The siRNA, negative control, was transfected into BMMs with LipofectamineTM 3000 (L3000015, thermofisher). The sequence of siRNA is as follows: sense (5'-3'): CGUAUUAUGAAAGUGGAGUAUTT, antisense (5'-3'): AUACUCCACUUUCAUAAUACGTT.

### Statistical analysis

The statistical analysis was conducted using GraphPad Prism software (version 8.0.2, San Diego, CA). The figures and legends provided information about the sample size for each genotype in each *in vivo* experiment. All *in vitro* experiments were performed with at least three independent replicates. The unpaired, two-tailed Student's t-test was used for statistical comparisons between the two groups. Multiple comparisons were assessed using an ordinary one-way ANOVA with Dunnett correction. All experimental data are expressed as the mean ± SD. A p-value < 0.05 is regarded as statistically significant.

## Supplementary Material

Supplementary figures and table.

## Abbreviations

OCs: Osteoclasts
BMMs: Bone marrow-derived macrophages
M-CSF: Macrophage colony-stimulating factor
RANKL: Receptor activators of nuclear factor kappa-Β ligand
TRAP: Tartrate-resistant acid phosphatase
OXPHOS: Oxidative phosphorylation
OCR: Oxygen consumption rate
Tfr1: Transferrin receptor 1
RIP: RNA-immunoprecipitation
ATP: Adenosine triphosphate
ROS: Reactive oxygen species
PMOP: postmenopausal osteoporosis patients
