# Supplementary material for: Rc3h1 negatively regulates osteoclastogenesis by limiting energy metabolism
Source: Theranostics. 2024 Nov 4;14(19):7554–68. doi: 10.7150/thno.99565 (PMC11626950; doi:10.7150/thno.99565)
Supplement: Supplementary file 1 — Supplementary figures and table. [file thnov14p7554s1.pdf]

Supplementary materials

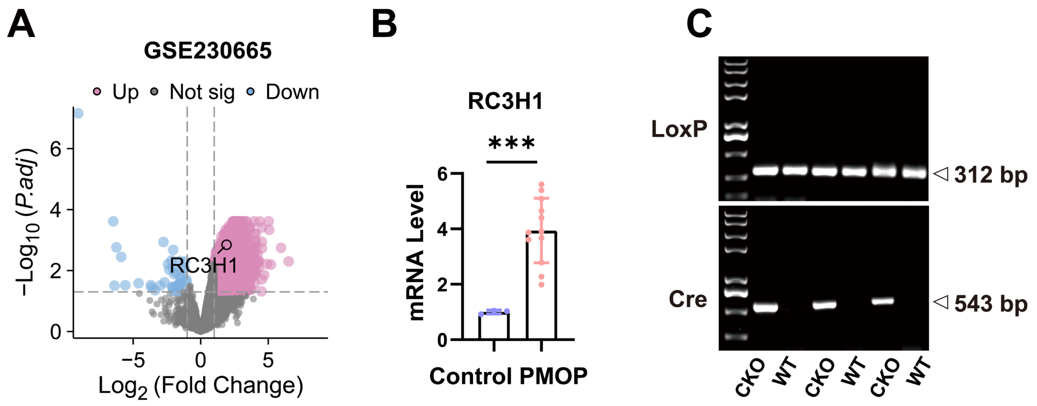

Figure S1. The expression of Rc3h1 is increased in bone tissues of PMOP patients. (A-B) Volcano plot and relative reads of Rc3h1 in control and PMOP patients form dataset GSE230665. PMOP: postmenopausal osteoporosis. (C) Identification of the genotype in Rc3h1 WT and CKO mice with DNA agarose gel electrophoresis. *P < 0.05, **P < 0.01, ***P < 0.001.

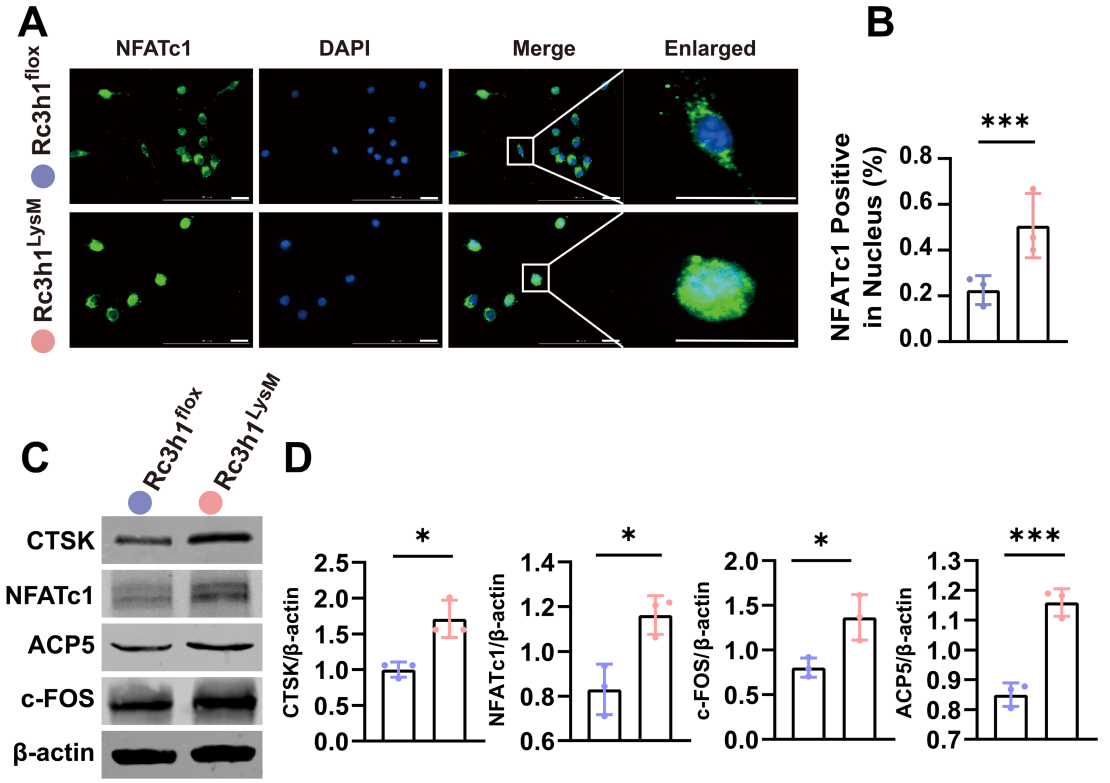

Figure S2. Rc3h1 negatively regulates osteoclast activation. (A) Immunofluorescence staining and statistical analysis of NFATc1 in the cytoplasm and nucleus of BMMs induced by M-CSF and RANKL for 48 hours. Scale bar= 20 µm. (C-D) WB images and quantitative analysis of CTSK, NFATc1, Acp5 and c-FOS in Rc3h1^{flox} and Rc3h1^{LysM} osteoclasts. *P < 0.05, **P < 0.01, ***P < 0.001.

Table S1 Primer sequence for osteoclast-related genes

Genes	Forward	Reverse
Acp5	ACGGCTACTTGCGGTTTCA	TCCTTGGGAGGCTGGTCTT
Dcstamp	TCTGCTGTATCGGCTCATCTC	ACTCCTTGGGTTCCTTGCTT
Ctsk	AGGCGGCTATATGACCACTG	TCTTCAGGGCTTTCTCGTTC
Fos	TACTACCATTCCCCAGCCGA	GCTGTCACCGTGGGGATAAA
Nfatc1	GGTGCTGTCTGGCCATAACT	GAAACGCTGGTACTGGCTTC
Tfrc	TTCGCAGGCCAGTGCTAGG	TACAAGGGAGTACCCCGACAG
Actb	TCTGCTGGAAGGTGGACAGT	CCTCTATGCCAACACAGTGC
